# Quality Management, Certification and Rating of Health Information on the Net with MedCERTAIN: Using a medPICS/RDF/XML metadata structure for implementing eHealth ethics and creating trust globally

**DOI:** 10.2196/jmir.2.suppl2.e1

**Published:** 2000-09-13

**Authors:** Gunther Eysenbach, Gabriel Yihune, Kristian Lampe, Phil Cross, Dan Brickley

**Affiliations:** ^1^Department of Clinical Social MedicineUnit for Cybermedicine & eHealthUniversity of HeidelbergGermany; ^2^FinOHTA / STAKES Finnish Office for Health Technology AssessmentFinland; ^3^ILRT Institute for Learning and Research TechnologyUnited Kingdom; ^4^W3C Metadata Interest GroupMassachusetts Institute for Technology (MIT)Boston MAUSA

## Abstract

MedCERTAIN (MedPICS Certification and Rating of Trustworthy Health Information on the Net, http://www.medcertain.org/) is a recently launched international project funded under the European Union's (EU) "Action Plan for safer use of the Internet". It provides a technical infrastructure and a conceptual basis for an international system of "quality seals", ratings and self-labelling of Internet health information, with the final aim to establish a global "trustmark" for networked health information. Digital "quality seals" are evaluative metadata (using standards such as PICS=Platform for Internet Content Selection, now being replaced by RDF/XML) assigned by trusted third-party raters. The project also enables and encourages self-labelling with descriptive metainformation by web authors. Together these measures will help consumers as well as professionals to identify high-quality information on the Internet. MedCERTAIN establishes a fully functional demonstrator for a self- and third-party rating system enabling consumers and professionals to filter harmful health information and to positively identify and select high quality information. We aim to provide a trustmark system which allows citizens to place greater confidence in networked information, to encourage health information providers to follow best practices guidelines such as the Washington eHealth Code of Ethics, to provide effective feedback and law enforcement channels to handle user complaints, and to stimulate medical societies to develop standard for patient information. The project further proposes and identifies standards for interoperability of rating and description services (such as libraries or national health portals) and fosters a worldwide collaboration to guide consumers to high-quality information on the web.

## Background

The evolution of the "information age" is mirrored in the exponential growth in the number of web sites and online accessible databases, and expanding services and publications available on the Internet [1]. Many consumers and patients directly apply the information they have read on the Internet to their own lives [2]. A well-known concern is the extremely variable quality of health related information on the Internet, which ranges from beneficial to harmful. Several studies have evaluated the quality of medical information on various venues of the Internet such as the World-Wide-Web [3], newsgroups [4] and email consultations [5]- [7]. As information technology and consumer health informatics are becoming integral parts of modern public health concepts and national health care policies in developed countries [8], implications of Internet information for public health are widely discussed topics [9,10].

Misinformation can lead patients with life-threatening conditions to lose trust in their provider, take actions that undermine the effectiveness of their treatment (e.g., by taking substances that interact in a negative way with prescribed medications). Patients may use their limited time with their healthcare provider unproductively in ways that ultimately increase costs of care, and even abandon a provider delivering high-quality care to pursue ineffective therapies. People with inadequate capabilities in critical thinking may also be victimized by biased or incomplete information from those with a financial interest in the information they provide [11].

Such risks are present in most media, but on the World-Wide-Web this problem reaches a new dimension. Therefore new technologies and services, which allow consumers to filter high-quality information, are needed to shift the balance towards more effective utilization of trustworthy and beneficial health information [8,12].

## Quality management of health information on the Internet

To realize the full potential of the Internet for self-help, self-care and patient empowerment, it is necessary to ensure the quality of information. As on the Internet any centralized body to assure quality is unrealistic and undesirable, one current challenge of consumer health informatics is to develop distributed applications that help consumers to assess the quality of information and to automatically filter information according to their needs [12].

Quality management of health information on the Internet essentially rests on four pillars - the four big E's [8,13]:

- **E**ducating consumers so that they are better able to identify and find good quality information
- **E**ncouraging health information publishers to self-regulation and self-labeling (disclosure, content description with metadata). This also involves educating publishers on ehealth ethics and best practice guidelines, so that they are better able to provide high quality information
- **E**valuating information by independent external third parties,
- **E**nforcement of existing legislation, in the case of fraudulent or harmful information.

We describe the implementation of these four pillars in an ongoing EU project named MedCERTAIN (MedPICS Certification and Rating of Trustworthy Health Information on the Net) [14]. This global project is funded by the European Union under the "EU Action Plan on promoting safer use of the Internet by combating illegal and harmful content on global networks" (http://www2.echo.lu/iap/). The Action Plan is pulling together academics, industry, consumers, and professional organizations in order to establish a comprehensive quality management system on the Internet, which includes a network of hot-lines, support for self-regulation, development of technical measures for rating and filtering, and awareness initiatives.

This paper will focus on the conceptual, organizational and technical framework of the MedCERTAIN project.

**Figure 1:**
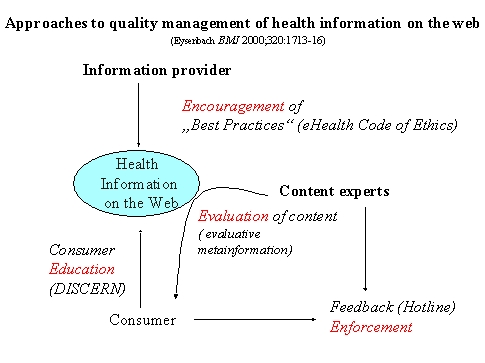
Approaches to quality management of health information on the web

## Overview

The technical and organisational infrastructure built by the project consortium will allow associations (e.g. medical societies) and individuals (e.g. medical domain experts) to rate (i.e. to assign metadata to) health information on the Net in a distributed, decentralised way, thus will allow consumers to put greater trust into reliable, evidence-based health information on the Net. The project will further establish a data exchange and interoperability structure allowing existing rating services, such as gateways, directories, libraries to contribute their evaluative metadata and to benefit from the existing data pool.

Health information providers will dynamically generated seals (trustmarks) showing their commitment to quality and the level of assessment the site has gone through. Consumers will be easily able to see which organizations have approved, accredited or recommended this site. Users will be able to access disclaimers, disclosure information and information on the editorial process in a standardized way, and will have standardized feedback channels to comment on the site. In addition, consumers will be able to use their browsers, or additional software or search engines to retrieve this metainformation automatically in the background whenever they access a website. The metadata will represent a free, "open directory", which can be shared freely and used for different applications. For example, search engines could use these data to rank search results. Similarly, health kiosks (publicly accessible Internet terminals for use in libraries and hospitals) may use this infrastructure to limit access to quality assessed content on the web, or to display disclaimers if the consumer is leaving the "evidence-based" (rated) subset of the web.

## Basic Concepts

*Expert ratings.* One basic principle of MedCERTAIN is the idea that only content experts can (and in fact have the duty to) guide patients to the best available information. Studies have shown that the formal and technical criteria that make a good website, such as user interface, disclosure, etc., do not correlate well with the quality of the content (in terms of accuracy). Technical criteria may generate trust and help to filter out bad information providers to a certain point, but does not allow to predict the accuracy of the content. Thus, the presence or absence of technical criteria such as the provision of references or the presence or absence of the HON-Logo are not sufficient markers for the public, instead we need content experts to guide consumers to the best available resources. Content experts are doctors and medical societies, and we think that these have the responsibility to take an active role in not only providing patient information themselves, but also in establishing standards for good patient information in their own fields.

*Decentralized, distributed rating.* Another principle of the MedCERTAIN project follows up from the idea that the quality of health information and interactive applications on the Internet cannot and should not be controlled by a central body or authority, but instead information and applications must be evaluated and be "labeled" in a decentralized manner [12,15,16]. Labeling means to provide meta-information, i.e. to provide information about information. We have previously pointed out that metainformation can be descriptive or evaluative. Descriptive metainformation is for example the name of the author, the subject area covered, and the date. Such information can be provided by the author using the Dublin Core metadata recommendation. However, specific requirements for health information, for example making clear sponsors or conflicts of interest, are not specified in the Dublin Core. Evaluative metainformation is for example information from external evaluators on whether the information on the website is accurate. To enable searching and filtering of information on the Internet, metainformation should use a standardized vocabulary. No such standardized vocabulary exists today. While Dublin Core Metadata provide some general (non medicine-specific) recommendations for metadata, no specific agreement on descriptive metadata exists today taking into account disclosure requirements of health information providers. Also for evaluative metadata no such standard vocabulary exists. The latter would require people to agree on a set of quality criteria that can be used for external evaluation of websites, the former would require to reach a consensus on disclosure requirements.

To fill this gap, we proposed (as early as in 1997) a medical core metadata vocabulary set based on the W3C PICS (Platform for Internet Content Selection) standard, which we called medPICS [17]. Proposed descriptive metadata of medPICS included authorship, qualification of authors, sources of funding, content keywords based on UMLS/MeSH etc [17]. Compared to other approaches, such as the provision of Dublin Core Metadata in HTML metatags, which allow only the use of descriptive metainformation supplied by webauthors [18], one idea behind medPICS was to make use of the fact that the PICS standard allows for metadata to be assigned by third parties (evaluative metadata) independently from the document, which can be used to evaluate (rate) information on other websites [12,17]. MedPICS therefore also contained a set of evaluative categories and was an early attempt to specify quality criteria that could be used by external evaluators.

One aim of MedCERTAIN is also to stimulate the more widespread use of descriptive metadata, whose uptake has been shown to be poor [19]. In contrast to other views on the use of metadata [18], we see the use of metadata not restricted to distribute descriptive elements to enhance retrieval, e.g by coding subject area, names of authors etc., but also to implement e-health ethics, i.e. to code disclosure information such as the qualification of authors, target group, sponsorship, attribution etc [17]., to allow consumers to view disclosure information in a standardized way, and to allow searching and filtering of information according to any of these criteria (e.g. "show me all websites about Alzheimer written by health professionals not sponsored by a pharmaceutical company"). In addition, a vocabulary of evaluative metadata should be agreed upon, which would to allow health portals, gateways, libraries, medical societies and other medical experts to describe in an evaluative way other health resources using attributes-value pairs such as "does the resource provide balanced information - yes/no".

The combination of descriptive and evaluative ratings can be used by consumers to filter information according to their needs and help them to make informed health decisions [20].

*Collaboration.* The third principle is global cooperation and interoperability, which is closely related to the concept of distributed and decentralized rating: if all health information rating services on the Internet would use a common, standardized "language" to describe and rate health information on the net, and if this data can be exchanged between the services and communicated to the consumer in a standardized way, then the user will have a huge resource of metainformation at his fingertips, helping him to assess the credibility of any health information he finds. The amount of information on the Internet is vast, and all attempts by any single institution to rate any information would be futile.

### Collaboration for Critical Appraisal of Health Information ("Heidelberg Collaboration")

We therefore need to build a global collaboration of individuals and organizations interested in enhancing the quality on the web, which involves to create standards and methods of providing health information to the public, to develop methods to evaluate health information and to create a communication infrastructure to share this metainformation ("*Collaboration for Critical Appraisal of Health Information*", or short "Heidelberg Collaboration"). We envisage that this collaboration, proposed already in 1997, [12] could get a similar role for consumer health information as the Cochrane Collaboration [21] has for information for professionals. While the Cochrane Collaboration attempts to extract and disseminate the scientific evidence from the scholarly literature, the Collaboration for Critical Appraisal of Health Information will extract and evaluate information that is provided to the public in the real world, primarily on the Internet, but also in other media. One of the benchmarks for evaluation will obviously be "evidence-basedness" (but also ethical and formal criteria of information provision), so that the Heidelberg Collaboration will build upon the work done by the Cochrane Collaboration. On the other hand, the Cochrane Collaboration and the evidence-based medicine movement will benefit from the work of the Heidelberg Collaboration, in that the Heidelberg Collaboration will help to disseminate evidence by guiding users to the best available information resources and in helping health information providers to improve their information. Moreover, comparing information that is actually provided to patients in the real world with information that should be provided to the public according to the standards of evidence-based medicine may help us to set priorities for research and systematic reviews, and to set priorities for health education and health promotion interventions in areas where obvious discrepancies between public knowledge and attitudes with the best available evidence exists.

Overall, it would be the main aim of the Heidelberg Collaboration to build a web of trust of responsible organisations and individuals who guide users to the sources on the Internet (and elsewhere) which provide evidence-based information presented in an ethically acceptable manner, to enable users to make evidence-based choices.

**Figure 2:**
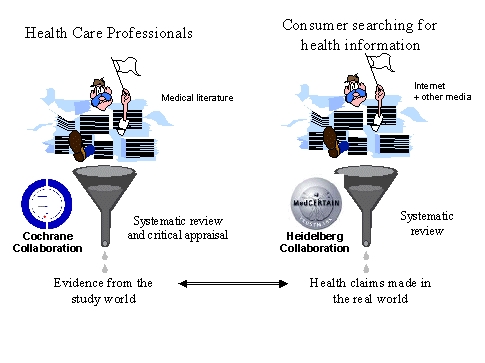
Just as the Cochrane Collaborations aims to appraise the medical literature for health professionals, the Heidelberg Collaboration appraises health information intended for the public

## Organizational framework for rating and labeling information

The MedCERTAIN consortium itself is not rating health information, but builds an organizational and technical infrastructure, which allows trusted individuals and organizations to evaluate information on other websites. The MedCERTAIN consortium also aims to create open standards for collaboration and interoperability among rating services. This evaluative metainformation coming from different sources, together with descriptive metainformation voluntarily assigned by the webauthors, can be used by consumers to filter high-quality information for their needs. Metainformation can either be provided by webauthors themselves or provided from a third-party rating service (or simply "label bureau").

## Approaches to deliver evaluative metainformation: First, second and third generation trustmarks

The most common approach to provide evaluative metainformation is "self-labeling" by means of putting a static "award" logo on a webpage to show endorsement by third parties (or to provide this information as text or by providing meta-tags). A problem of these "first generation" approaches is that such awards and logos can be included by webmasters themselves and therefore are more suited for marketing purposes rather than to provide reliable reassurance to consumers.

Second generation approaches make such logos or awards "clickable" and allow consumers to check the current standing of a website by directly linking to the third-party site which would display a dynamic record. Examples for services using this approach are VeriSign, E-trust, or VIPPS (Verified Internet Practice Sites) of the National Association of Boards of Pharmacy). The Health on the Net (HON) Logo [22] is currently transiting from a first- to a second generation approach in now asking information providers to put a hyperlink over their logo which leads back to the HON site.

Both first and second-generation approaches allow webauthors to self-publish "awards" or evaluative metainformation selectively, with no possibilities for rating services to bring this information directly to consumers. While such approaches make sense for descriptive metainformation (e.g. information about the authors, date of publication, qualification of authors etc [17,18]. provided in metatags) they are problematic for evaluative metainformation.

Third generation approaches would allow consumers to directly retrieve metainformation from one or more rating services in real time while accessing a website.

**Figure 3a:**
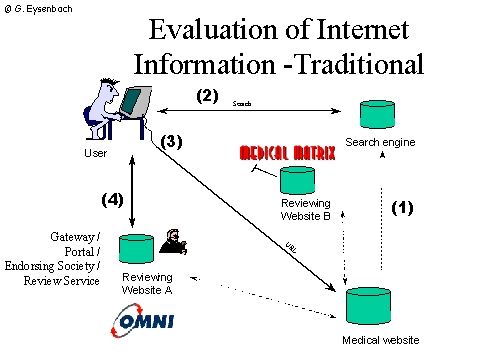
The drawback of the traditional model of having portal sites is that consumers have to browse to one of these portals explicitely to get the ratings. Also, they don't know what rating service B has to say if they are on rating service A. Moreover, if the end up directly on a website (e.g. via a search engine), they don't know what rating service A or B have to say about this website

**Figure 3b:**
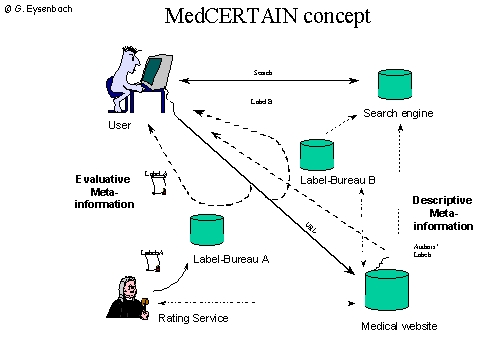
The medPICS standard can be used to transmit metainformation directly to consumers, for example to label excellent or fraudulent websites.The browser of the user automatically requests labels (metainformation) from different services

**Figure 4:**
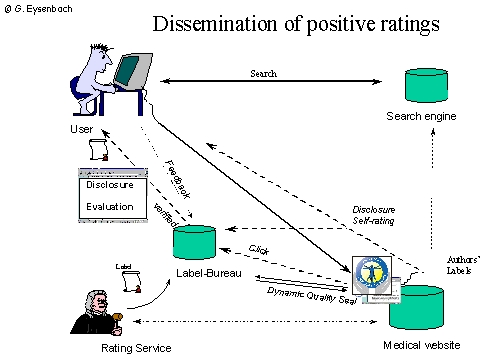
Dynamic quality seals, which are linked to a dynamic MedCERTAIN page, providing additional descriptive and evaluative information about the website, can be displayed by trustworthy health websites

Two complementary approaches will be tested for the MedCERTAIN project to convey evaluative metainformation to consumers:

- PICS (RDF/XML) labels (Fig. 3b), which are invisible for the user and require client software (such as the web browser) to display the ratings.
- Dynamic quality "seals" (Fig. 4), which can be included by websites which have been positively rated:

## PICS (RDF/XML) labels

The advantage of the medPICS approach [16] is that consumers can receive PICS (RDF/XML) labels (Fig. 3) (metadata) directly from a third-party label bureau, without the rated website having to co-operate or having the possibility to tamper with the rating. This is a pre-requisite for being able to deliver critical or disapproving comments, in extreme cases they would allow "blacklisting" harmful websites, such as websites making fraudulent health claims, by appropriate bodies such as the FTC (US Federal Trade Commission).

## Technical discourse: From PICS to RDF/XML

The PICS standard (http://www.w3.org/PICS/) is currently migrating towards becoming an application of the XML/RDF technology of the W3C (see http://www.w3.org/RDF/). The successor standard, RDF (the Resource Description Framework), grew out of work on expanding PICS (then called PICS-NG) to provide for more flexible descriptive capabilities (e.g. textual comments). RDF is a W3C Recommendation for Web "resource description" which includes labeling, classification, cataloguing, rating etc. RDF in turn adopts the W3C XML Recommendation as a new file format for exchanging such data, replacing the PICS 1.1 format.

We continue to call our vocabulary to describe health resources medPICS rather than moving to a new terminology such as medRDF. The MedPICS demo version 0.3 [17] will be replaced by the MedPICS consensus version 1.0 (to be developed and agreed upon in the Heidelberg workshop in September 2000), which will be expressed in RDF. This will be the first consensus metadata schema for health information on the Internet, designed with practical application within the MedCERTAIN project in mind.

### Dynamic quality "seals"

Dynamic third-generation quality "seals" in form of dynamically (on-the-fly) created jpeg or gif logos (Fig. 3) are primarily useful for "whitelisting" and labeling trustworthy health websites. In the MedCERTAIN project, they will - in addition to medPICS metadata - be used for example by health information providers to indicate that they have committed themselves (in writing) to stick to a consensus e-health Code of Ethics [23], are in "good standing", and volunteer to disclose certain information, e.g. authorship, sources of funding, internal quality control mechanisms etc. On higher levels of evaluation they would also indicate that they have for example been checked or accredited by medical societies.

The web publisher includes an IMG SRC tag in his HTML code which points to a remote server (the MedCERTAIN server), which will create a seal in real time (based on information found in the MedCERTAIN database) and sends it to the user. The "seal" can therefore incorporate current information from the MedCERTAIN database, for example the logos of the societies endorsing the website (as animated gif), a timestamp and the URL of the site. If important information changes (for example, if the level of certification changes), it is not the responsibility of the webmaster of the health information provider, to remove or change the logo, but this will be done automatically. In addition, users still can (similarly to the second generation approach) click the logo to display further information, such as the disclosure information (provided by the health information provider and stored in the MedCERTAIN database), narrative rating information provided by professional raters or feedback comments from other users.

Both approaches - MedPICS metadata labels (which rely on the client software to display the information) and dynamic quality seals (which do not require any special set up by users) - can be used simultaneously and are not mutually exclusive. Evaluative medPICS labels and dynamic quality seals are generated from the same database containing evaluative metainformation about the website in question. Both approaches have the advantage compared to first- and second generation labels that information is send directly to the users without giving webmasters the possibility to tamper with the ratings. Third generation trustmarks are a prerequisite for external evaluations and monitoring of sites.

## Levels of evaluation and accreditation

The MedCERTAIN consortium is currently considering to use the following four different levels of certification for publishers of health information on the web. The levels build upon each other, which means that a health information providers has first to get through a level-1 certification process in order to get evaluated on level-2 or level-3 and to carry the MedCERTAIN trustmark indicating the respective level.

- Level 1 labels ("*committed"*) indicate that the site is in "good standing" (no complaints are filed), that the information provider has agreed in writing to follow an consensus E-health Code of Ethics [23] and that basic disclosure information has been submitted. This disclosure information (which also contains basic information such as the address of the provider, verified by MedCERTAIN, or editorial, privacy and advertising policies of the provider) can be retrieved by the consumer either by clicking on the seal (a new webpage opens, generated by the MedCERTAIN site) in narative form, or by means of medPICS metainformation in form of coded, computer-readable statements.
- Level 2 labels ("*checked"*) indicate that the website has been checked by a third party (a member of the MedCERTAIN collaboration) and fulfills the formal and technical criteria, specifically the criteria outlined in the Washington eHealth Code of Ethics
- Level 3a labels ("*awarded"*) indicate that the content of a website has been evaluated in terms of accuracy (including currency and completeness) and endorsed by an external individual rater (expert) or a rating organisation, such as a medical society, who are content experts. Based on these evaluations the rater would make a general judgement on the trustworthiness of the publisher.
- Level 3b labels (*"peer-reviewed"*) indicate that the content of a document (a webpage) has been peer-reviewed by an independent third party. This level will only be used in special cases (such as clinical guidelines) and also involves that the content needs to be re-evaluated if significant chances have been made.
- Level 4 labels (*"evidence-based"*) will indicate that the effectiveness of the ehealth service has been evaluated in a observational or experimental study, i.e the positive outcome of consumers or patients using the site, satisfaction, knowledge or behavior change, impact on mortality, morbidity or quality of life has been demonstrated in a scientific study and published in a peer-reviewed journal. This label only would apply to certain types of ehealth services and will in practice be rarely awarded. One implicit aim of preparing this level is to encourage health information providers to conduct, enable or solicit formal studies of the effectiveness of their services.

In summary, level 1 and level 2 evaluations check compliance with structural quality criteria, level 3 evaluate the process (of giving advice and support to users), and level 4 refers to the outcome.

**Figure 5:**
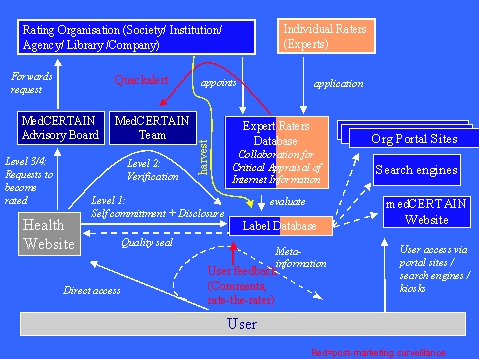
Organisational framework for a collaborative rating structure

## Who rates the information?

We envisage two possible avenues for rating: Individual expert raters and rating organisations.

Individual experts probably need to get reimbursed for their time and effort they invest. Contrary to the peer-review process in professional journals, we do not have a culture that experts review patient information free of charge and without getting any credits. In the "age of the patient", this culture may change at some point.

In the case of organisations we primarily envisage medical societies, whose aims should also be patient education. These organisations may decide whether they offer evaluations for free (as part of their statutory mission) or whether they charge information providers.

A number of organizations, institutions and individual experts are already in the business of evaluating, accrediting, or endorsing information. Some (for example professional societies) are simply publishing links as endorsements on their websites, others (such as Medical Matrix) maintain databases with evaluative information. The problem is that all these efforts are made in a non-coordinated way and that no common rating criteria are used. Many of the rating criteria commonly in use are not even validated [24].

## Goal: Interoperability of rating services

A basic idea of MedCERTAIN is to foster cooperation and interoperability of these services. If all health information rating services on the Internet would use a common, standardized "language" (evaluative metadata) to describe and rate health information on the net, and if this data can be exchanged and communicated to the consumer in a standardized way, then the user will have a huge resource of metainformation at his fingertips, helping him to assess the credibility of any health information he finds.

**Figure 6:**
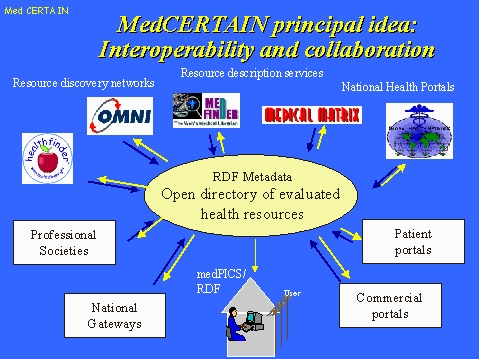
A standard that ensures interoperability of rating services would enable data exchange between rating services and allow the consumer to access many different "views"

## Consensus rating criteria for collaborative evaluation

In September 2000 a group of experts will gather in Heidelberg, to draft the "Heidelberg recommendations", which contain a basic set of reliable internationally accepted consensus quality criteria for health related websites, that can be used for assessment by a third party. This includes a rating vocabulary (a computer-readable representation of these rating categories, expressed as attributes [properties] and their possible values); a set of descriptive metadata categories, which will allow health websites to disclose essential information required in the Washington Code of eHealth Ethics [23] in a standardized, computer-readable way; and a data exchange structure which assures interoperability of rating services. A "Collaboration for Critical Appraisal of Health Information", proposed already in 1997, [12] is currently being formed, which brings together organisations and individuals who are active in the field of reviewing, appraising, rating, evaluating health information on the web, based on the consensus criteria, and to further develop methods and to exchange data. Together with the Cochrane Collaboration, this initiative hopes to improve dissemination of evidence to consumers on the Internet and thereby to advance evidence-based decision making in health care in empowering consumers to make informed, evidence-based decisions.

## Acquisition of ratings

A rating organisation can be for example a trusted medical society who chooses to certify websites relevant to their work, or a library attempting to collect high-quality resources on the web.

If a publisher wishes a level-3 or level-4 certification from any or a specific society or body he would contact the MedCERTAIN collaboration, which would forward the rating request to the respective society/body (Members of the MedCERTAIN collaboration can decide whether they rate information for free or whether they charge a fee to the publisher).

Raters are individual experts, working either independently or are affiliated with a rating service/organisation. The MedCERTAIN rater database currently contains more than 100 experts, who voluntarily or for a fee evaluate health information on the Net.

Rating services, such as professional societies or libraries, collaborating with the MedCERTAIN project, can supply ratings through two different venues: Either, rating services publish their metainformation in XML on their site, and the MedCERTAIN database will automatically harvest these ratings, or a rating service (such as a professional society wishing to endorse a website) uses tools which will be supplied by the MedCERTAIN consortium, such as bookmarklets, the RDF whatis-related menu, the HTML forms interface, bookmark uploads, remote bookmark storage (in LDAP repositories or JAVA-Applets. Digital signatures may ensure the authenticity of these ratings.

**Figure 7:**
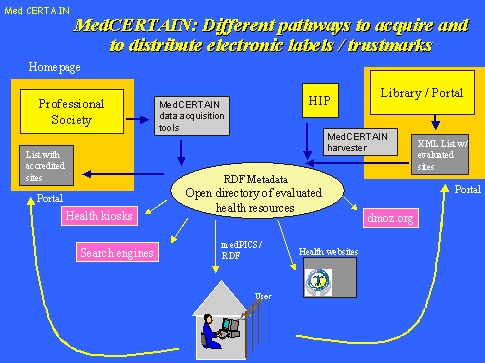
Labels (ratings) can be either acquired using MedCERTAIN tools (left) or can be published on the homepage of a collaborating portal using a standard XML vocabulary, from which it will be harvested by the MedCERTAIN robot. Once acquired, labels will be redistributed for example for health kiosks (enabling filtered access to a qualitative acceptable subset of the Internet), search engines (enabling ranking by quality), medPICS (directly to the consumer), open directories such as dmoz.org or will be used to dynamically create "seals" (logos) on the health information provider's homepage

Once acquired, labels will be redistributed for example for health kiosks (enabling filtered access to a qualitative acceptable subset of the Internet), search engines (enabling ranking by quality), medPICS (directly to the consumer), open directories such as dmoz.org or will be used to dynamically create "seals" (logos) on the health information provider's homepage

## How does the metadata come to the user?

The ratings gathered in the MedCERTAIN database constitute a RDF "open directory" will be redistributed and can reach the consumer through different channels. With PICS technology, consumers will be able to use their browsers, or additional software, to retrieve this metainformation automatically in the background whenever they access a website.

Similarly, health kiosks (publicly accessible Internet terminals for use in libraries and hospitals) may use this infrastructure to limit access to quality assessed content on the web, or to display disclaimers if the consumer is leaving the "evidence-based" (rated) subset of the web.

An important aspect will be that MedCERTAIN partners with search engines and will feed RDF metadata into these databases. This will allow search engines to rank their search results according to quality.

In addition, the collaborating rating services (e.g. medical societies) may publish this information on their site, by dynamically accessing the open directory to compile a subject-specific directory on their pages.

Finally, health information providers themselves may publish a dynamically generated seal described above which will make visible relevant information from the MedCERTAIN database to users when they access the website.

Although funded by the European Union, it should be emphasized that the project is not restricted to Europe as an geographical area, but aims to provide a global infrastructure. Existing organization who are active in this field have been invited to participate, including the Health in the Net Foundation, URAC and the Internet Health Coalition.

In summary, this ambitious project attempts to create a global infrastructure for health metainformation, using the connectivity and decentralized of the web to exchange information about information, essentially to build a "web of trust", which is able to direct and guide consumers to accurate and relevant health resources.
